# Inferior Vena Cava Graft–Enteric Fistula Presenting with Occult Gastrointestinal Bleeding and Psoas Abscess: A Rare Case Report

**DOI:** 10.3390/jcm15156002

**Published:** 2026-08-02

**Authors:** Chuwen Chen, Lijia Wei, Yiyuan Li, Bin Huang, Xiyang Chen

**Affiliations:** Division of Vascular Surgery, Department of General Surgery, West China Hospital, Sichuan University, Chengdu 610041, China; chenchuwen@scu.edu.cn (C.C.); weilijiaaa@163.com (L.W.); liyiyuan991101@126.com (Y.L.); hxyyhhuangbin@126.com (B.H.)

**Keywords:** inferior vena cava, graft enteric fistula, duodenal erosion, vascular graft infection, psoas abscess, occult gastrointestinal bleeding

## Abstract

**Background**: Inferior vena cava (IVC) graft–enteric fistula is an exceptionally rare but potentially fatal complication of caval reconstruction. **Case Presentation**: A 22-year-old woman with previous IVC and right renal vein reconstruction presented with an 18-month history of intermittent abdominal pain, diarrhea, and progressive anemia. On admission, contrast-enhanced computed tomography demonstrated a thrombosed IVC graft with perigraft gas and fluid, loss of the fat plane between the graft and adjacent bowel, and a multiloculated right psoas abscess. *Escherichia coli* was isolated from blood and drainage cultures. Upper gastrointestinal endoscopy directly visualized prosthetic graft material protruding into the descending duodenum, confirming a graft–enteric fistula. After targeted antimicrobial therapy and stabilization, the patient underwent complete graft explantation, resection of the involved duodenal and jejunal segments, duodenojejunal reconstruction, and extensive retroperitoneal debridement. She remained free of recurrent infection, fistula, and gastrointestinal bleeding at 5-year follow-up. **Conclusions**: In patients with previous caval reconstruction, persistent gastrointestinal symptoms, unexplained anemia, bacteremia, or retroperitoneal infection should raise suspicion for a graft–enteric fistula. Computed tomography may identify suggestive perigraft changes, whereas endoscopy can provide direct confirmation of luminal erosion.

## 1. Introduction

Vascular grafts are widely used in the repair of complex vascular injuries, vascular reconstruction following tumor resection, and the treatment of major vascular diseases. They play a critical role in restoring blood flow and maintaining vascular continuity. With advances in vascular reconstruction techniques and improved long-term patient survival, increasing attention has been directed toward late graft-related complications. These complications include graft thrombosis, infection, and erosion into adjacent structures. Although vascular graft–enteric fistula is rare, it is potentially life-threatening and may result in severe infection, gastrointestinal bleeding, or death [1]. Secondary arterioenteric fistulas, particularly those occurring after abdominal aortic reconstruction, have been well documented [2,3,4,5,6]. They typically involve bowel segments in close proximity to major vessels, most commonly the duodenum, esophagus, and sigmoid colon [7,8]. Their development is generally associated with graft infection, chronic local inflammation, mechanical compression, and progressive erosion into the adjacent bowel [9]. In contrast, venous graft–enteric fistulas are exceedingly rare [9,10]. This may be attributable to the lower intraluminal pressure and distinct hemodynamic characteristics of the venous system, as well as the relatively limited mechanical stress exerted by venous grafts on adjacent tissues. The diagnosis of a venous graft–enteric fistula is challenging because early clinical manifestations are often nonspecific and imaging findings may lack specificity. To date, only a few cases of duodenocaval fistula following inferior vena cava (IVC) stent placement have been reported [11], whereas similar complications involving prosthetic caval grafts remain exceedingly rare. Nevertheless, this potentially life-threatening complication should be considered in patients with a history of IVC reconstruction who present with otherwise unexplained infection, abdominal symptoms, or gastrointestinal manifestations. We report a rare case of an IVC graft–enteric fistula and describe its clinical presentation, diagnostic evaluation, and surgical management, with particular emphasis on the radiographic, endoscopic, and intraoperative findings. This case underscores the importance of maintaining a high index of suspicion for this uncommon but serious complication.

## 2. Case Presentation

A 22-year-old woman presented with recurrent lower abdominal discomfort and intermittent diarrhea. She had no history of hypertension, diabetes mellitus, or other chronic disease and was not taking long-term medications or herbal supplements. She was unmarried and not pregnant and denied smoking, alcohol consumption, recreational drug use, recent travel, or unusual environmental exposure. Her family history was unremarkable, with no known hereditary disorders.

Thirty months before admission, she sustained injuries to the IVC and right renal vein in a motor vehicle accident and underwent emergency surgery at a local hospital. A Dacron graft (Gelweave^TM^; Terumo Aortic, Vascutek Ltd., Inchinnan, UK) was used for in situ IVC and right renal vein reconstruction. Retroperitoneal soft tissue injury was noted, but no bowel injury was identified. Postoperatively, rivaroxaban, 20 mg once daily, was initiated.

At 3-month follow-up, imaging revealed thrombosis of the IVC graft extending from above the renal veins to the bilateral common iliac veins. The patient had no lower extremity edema, venous congestion, renal dysfunction, or other symptoms of impaired venous return. Given the absence of venous outflow obstruction, adequate collateral circulation, long-segment occlusion, and high risk of reoperation, conservative management was selected. No graft recanalization or repeat venous reconstruction was performed, and rivaroxaban was continued with regular clinical and imaging follow-up.

Approximately 12 months after vascular reconstruction, she developed recurrent dull lower abdominal pain with intermittent diarrhea. The pain was non-radiating, unrelated to food intake, and rated 2 on the visual analog scale (VAS). Diarrhea occurred 3 to 4 times daily and consisted of loose yellow stools. She denied nausea, vomiting, melena, or cessation of flatus, and her appetite, sleep, and body weight remained stable. Evaluation at a local hospital showed mild normocytic anemia, with a hemoglobin level of 112 g/L, and mildly elevated inflammatory markers. Leukocyte and platelet counts, as well as liver and renal function, were normal. Contrast-enhanced computed tomography (CT) demonstrated chronic IVC graft thrombosis without progression, perigraft abscess, or definite bowel abnormality. Colonoscopy revealed only mild focal mucosal erythema and edema and several colonic polyps. Symptomatic treatment provided temporary relief, but symptoms recurred intermittently.

**Figure 1:**
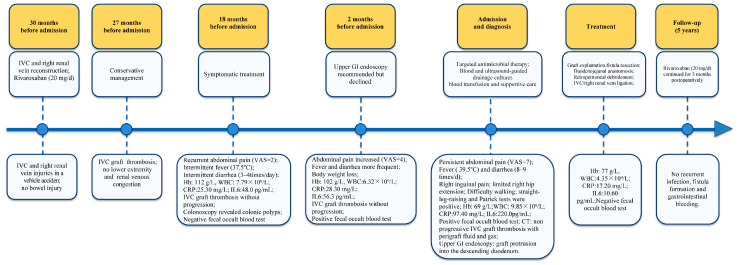
**Flowchart illustration depicting the medical timeline of this** **patient.**

Two months before admission, her symptoms worsened, with increased abdominal pain (VAS score, 4), more frequent fever, diarrhea 5 to 6 times daily, decreased appetite, and an unintentional weight loss of approximately 2 kg. Repeat laboratory testing showed worsening anemia, with a hemoglobin level of 102 g/L, elevated inflammatory markers, and intermittently positive fecal occult blood tests. Repeat CT again showed chronic IVC graft thrombosis without progression but no definite infectious focus or fistula. Upper gastrointestinal endoscopy was recommended because of persistent gastrointestinal symptoms and positive fecal occult blood testing, but the patient declined. Ten days before admission, she developed persistent right lower quadrant pain, with a VAS score of 7, high-grade fever up to 39.5 °C, and diarrhea 8 to 9 times daily. She also developed limited right hip extension, right inguinal pain, and difficulty walking. Physical examination revealed deep tenderness in the right lower quadrant without rebound tenderness or guarding. Straight-leg-raising and Patrick tests were positive. The patient’s clinical timeline was shown in Figure 1.

**Figure 2:**
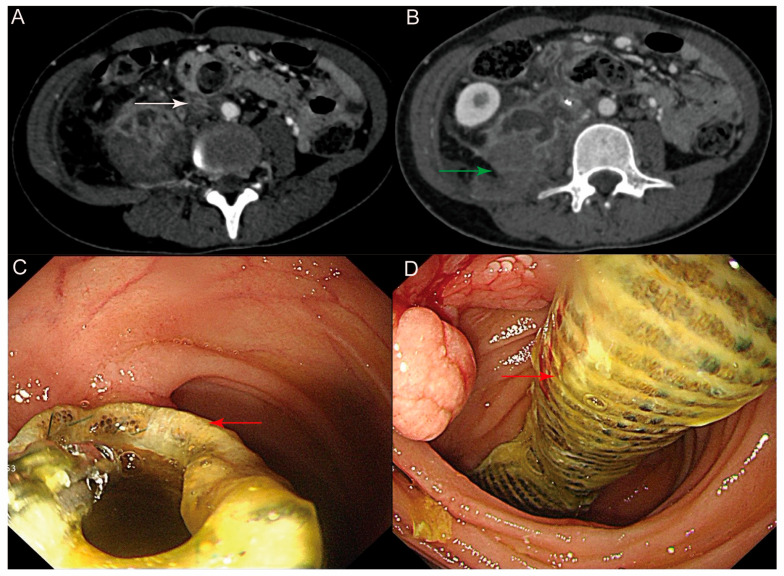
**Diagnostic imaging and endoscopic findings.** (**A**) Contrast-enhanced computed tomography showing loss of the fat plane between the inferior vena cava graft and adjacent bowel (white arrow); (**B**) multiloculated right psoas abscess (green arrow); (**C**) upper gastrointestinal endoscopy showing the proximal graft segment protruding into the descending duodenum (red arrow); (**D**) prosthetic graft material exposed within the duodenal lumen, confirming graft enteric fistulization (red arrow).

On admission, laboratory studies showed severe anemia, with a hemoglobin level of 69 g/L; leukocytosis, with a leukocyte count of 9.85 × 10^9^/L; markedly elevated C-reactive protein, 97.40 mg/L; interleukin-6, 220.0 pg/mL; and an erythrocyte sedimentation rate of 84 mm/h. Fecal occult blood testing was strongly positive. Emergency contrast-enhanced CT demonstrated a non-opacified IVC graft with surrounding fluid and gas collections and loss of the normal fat plane between the graft and adjacent bowel (Figure 2A). A multiloculated abscess was also present in the right psoas and iliopsoas muscles (Figure 2B). These findings suggested graft infection with enteric fistulization. Blood cultures and ultrasound-guided drainage cultures both grew *Escherichia coli*. Upper gastrointestinal endoscopy subsequently showed direct erosion and protrusion of the prosthetic graft into the descending duodenum, with extensive intraluminal exposure of graft material (Figure 2C,D), confirming the diagnosis of an IVC graft–enteric fistula.

**Figure 3:**
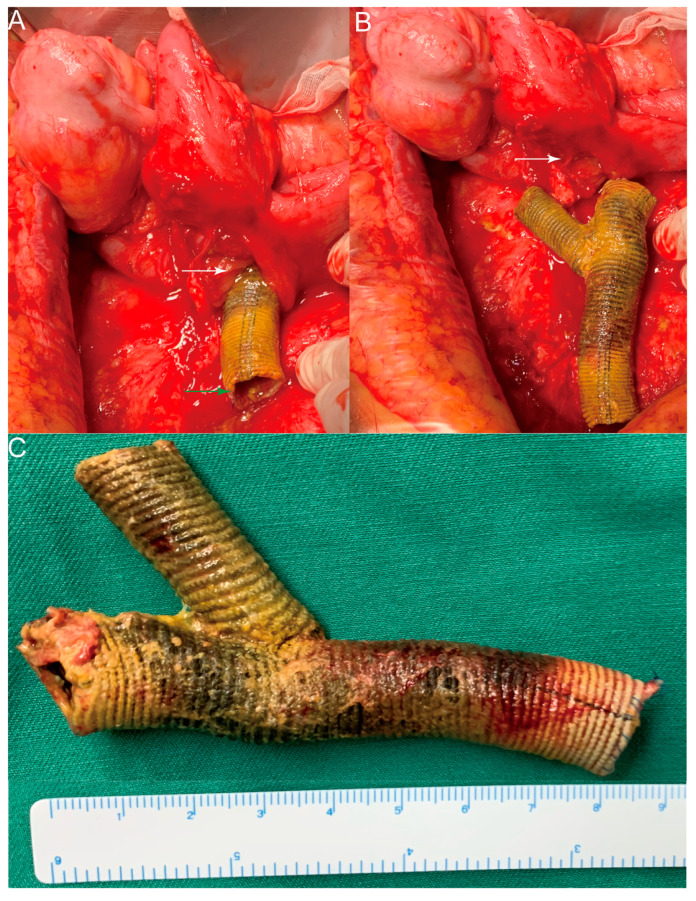
**Intraoperative findings and explanted inferior vena cava graft**. (**A**) Intraoperative view showing erosion of the proximal graft into the duodenum with surrounding inflammation and purulent material (white arrow), together with disruption of the distal anastomosis (green arrow); (**B**) direct communication between the prosthetic graft and duodenal lumen through the fistulous tract (white arrow); (**C**) explanted bifurcated polypropylene graft with dark discoloration and adherent inflammatory debris, consistent with chronic infection and enteric erosion.

Targeted antimicrobial therapy with imipenem, 1 g every 8 h, was administered for 7 days according to susceptibility testing, together with blood transfusion and supportive care. After substantial improvement in inflammatory markers, exploratory laparotomy was performed. Dense adhesions were found among the duodenum, retroperitoneum, and IVC graft. The graft was completely occluded by organized thrombus and had partially eroded into the duodenal lumen (Figure 3A,B). A jejunal fistula and extensive retroperitoneal abscess extending to the right renal hilum and psoas muscle were also identified. Erosive changes were present at all anastomotic sites (Figure 3C).

Definitive management included complete graft explantation, resection of the duodenal and jejunal fistulas, duodenojejunal anastomosis, and extensive retroperitoneal debridement with drainage. Because the proximal and distal IVC segments connected to the graft and the right renal vein were chronically thrombosed, and because the patient had no symptoms of lower extremity or right renal venous hypertension, collateral venous compensation was considered sufficient. Therefore, the proximal and distal IVC and right renal vein were ligated. Low-molecular-weight heparin was started on postoperative day 2 for thromboprophylaxis. After discharge, the patient was transitioned to rivaroxaban, 20 mg once daily, which was discontinued 3 months after surgery. Histopathological examination showed chronic inflammation and mucosal erosion of the involved bowel segments, with marked inflammatory infiltration around the graft material. The postoperative course was uneventful. Fever resolved promptly, and gastrointestinal and musculoskeletal symptoms completely subsided. At 5-year follow-up, the patient remained asymptomatic, with no recurrent infection, fistula formation, or gastrointestinal bleeding.

## 3. Discussion

Vascular graft enteric fistula was a rare but life-threatening complication following vascular reconstruction, most commonly reported after aortic surgery [5]. In contrast, fistulas involving venous grafts, particularly after IVC reconstruction, were exceedingly uncommon and remain poorly characterized [12]. The present case highlighted an unusual manifestation of graft enteric fistula arising from a thrombosed IVC prosthesis, emphasizing the diagnostic challenges and clinical implications associated with this entity. Compared with arterioenteric fistulas, which often presented with overt gastrointestinal hemorrhage, venous graft enteric fistulas tended to follow a more indolent clinical course. In this case, the patient initially exhibited nonspecific symptoms, including intermittent abdominal pain, low-grade fever, and diarrhea, which persisted for an extended period before diagnosis. The absence of massive bleeding could be explained by the lower intraluminal pressure within the venous system, which might have delayed the onset of catastrophic hemorrhage and contributed to a more insidious presentation. As a result, diagnosis was often delayed, and the condition might have been overlooked until advanced complications such as sepsis or abscess formation occurred.

Another important feature in this case was the presence of a psoas and iliopsoas abscess, which manifested clinically as difficulty extending the hip and a positive FABER test. These findings reflected retroperitoneal extension of infection, a pattern that had been reported in association with vascular graft infections but was not commonly recognized as an early indicator of graft enteric fistulization. Clinicians should therefore have maintained a high index of suspicion when musculoskeletal symptoms coexisted with systemic inflammatory signs in patients with a history of vascular reconstruction. The diagnostic pathway in this case highlights the complementary roles of CT and endoscopy. Contrast-enhanced CT is useful as the initial imaging modality and may show suggestive findings, including perigraft gas, perigraft fluid, soft-tissue inflammation, abscess formation, and loss of the normal fat plane between the graft and adjacent bowel [7,8,13,14]. However, these findings may not definitively demonstrate a fistulous communication. In the present case, upper gastrointestinal endoscopy provided direct confirmation by showing prosthetic graft material protruding into the descending duodenum. Therefore, when CT findings are suspicious but not diagnostic, endoscopy should be considered, particularly in patients with unexplained gastrointestinal symptoms, occult bleeding, or persistent infection after vascular reconstruction [15,16].

The published cases summarized in Table 1 further demonstrate the heterogeneity and severity of IVC graft/stent–enteric fistulas. Most reported fistulas involved the duodenum, likely reflecting its close anatomic relationship with the infrarenal or suprarenal IVC. Initial reconstruction methods varied, including ePTFE or Dacron graft replacement and endovascular stent-graft placement. Clinical presentations ranged from gastrointestinal bleeding and anemia to recurrent sepsis, bacteremia, abscess formation, and bowel obstruction, explaining why diagnosis can be difficult when overt hemorrhage is absent. Management should be individualized according to the patient’s clinical status, graft patency, collateral venous drainage, and extent of infection. Most successfully treated cases required definitive source control, including removal of infected prosthetic material, repair or resection of the involved bowel, and either IVC ligation or selective reconstruction. Conservative management has been rarely reported and may be feasible only in highly selected patients with chronic graft occlusion, adequate collateral drainage, and limited infectious burden. Overall, reported outcomes suggest that prompt surgical source control is associated with favorable recovery, whereas persistent or recurrent infection portends a poor prognosis. These findings underscore the importance of early recognition, multidisciplinary planning, and long-term surveillance after caval reconstruction.

Several limitations should be acknowledged. As a single case report, the findings may not be generalizable. In addition, the exact timeline of fistula formation could not be determined, and earlier endoscopic evaluation might have facilitated a more timely diagnosis. Nevertheless, the detailed clinical course and long-term follow-up provide valuable insights into this rare condition.

## 4. Conclusions

Venous graft–enteric fistula after IVC reconstruction is exceedingly rare but potentially fatal. In patients with prior caval reconstruction, unexplained gastrointestinal symptoms, occult bleeding, persistent infection, or retroperitoneal abscess should prompt suspicion for graft–enteric fistula. CT findings such as perigraft gas, fluid collection, abscess formation, and loss of the fat plane between the graft and bowel are important warning signs, while endoscopy may provide direct diagnostic confirmation. Early multidisciplinary evaluation and definitive surgical source control are essential for favorable outcomes.

## Figures and Tables

**Table 1:** **Published cases of inferior vena cava graft/stent–enteric fistula after caval** **reconstruction.**

Author-Year	Reconstruction Procedure	Fistula Site	Main Presentation	Diagnostic Method	IVC Reconstruction	Outcome
Nicolo et al. 1990 [17]	ePTFE graft	Duodenum	Paraprosthetic infection; septic; thrombosis	NR	NR	NR
Perera et al. 2004 [12]	ePTFE graft	Duodenum	Upper gastrointestinal hemorrhage	CT and EGD	PTFE graft	Recurrent fistula and death
Hamblin et al. 2011 [18]	stent-grafts	Duodenum	Sepsis; massive lower gastrointestinal bleeding	CT and cavography	Stent-graft	Recurrent sepsis and death
Addeo et al. 2012 [9]	ePTFE graft	Biliary loop	Recurrent sepsis	CT	IVC ligation	Successful treatment
Ippolito et al. 2015 [19]	ePTFE graft	Duodenum	Vomiting, abdominal pain, small-bowel obstruction	CT	Conservative	asymptomatic
Jeng et al. 2015 [20]	stent-grafts	Duodenum	Persistent sepsis	CT and cavography	IVC ligation	Successful treatment
Türkoğlu et al. 2015 [21]	Dacron graft	Duodenum	Hematemesis and melena	CT and EGD	IVC ligation	Successful treatment
Mahmood et al. 2016 [22]	Dacron graft	Duodenum	Melena, symptomatic anemia, and right-sided abdominal pain	CT and EGD	IVC ligation	Successful treatment
Rabindran et al. 2016 [23]	ePTFE graft	Duodenum	sepsis	CT and EGD	IVC ligation	Successful treatment
Hinojosa et al. 2021 [24]	ePTFE graft	Duodenum	gastrointestinal bleeding and bacteremia	CT and EGD	IVC ligation	Successful treatment
Koval et al. 2023 [25]	stent-grafts	Duodenum	Recurrent bacteremia	CT and PET-CT	IVC ligation,	Successful treatment
Wang et al. 2025 [26]	stent-grafts	Duodenum	Abdominal pain, septic shock, bacteremia, and fungemia	CT and EGD	IVC ligation	Successful treatment
Heuvelings et al. 2026 [11]	stent-grafts	Duodenum	Fever, polymicrobial bacteremia	CT and EGD	IVC ligation	Successful treatment

IVC, inferior vena cava; ePTFE, expanded polytetrafluoroethylene; PTFE, polytetrafluoroethylene; CT, computed tomography; EGD, esophagogastroduodenoscopy; PET-CT, positron emission tomography–computed tomography; NR, not reported. “Successful treatment” indicates clinical improvement or survival without early recurrence as reported in the original article.

## Data Availability

The datasets analyzed during the current study were available from the corresponding author upon reasonable request.
